# Childhood skeletal lesions common in prehistory are present in living forager-farmers and predict adult markers of immune function

**DOI:** 10.1126/sciadv.adw3697

**Published:** 2025-07-16

**Authors:** Amy S. Anderson, Aaron Blackwell, M. Linda Sutherland, Thomas Kraft, James Sutherland, Bret Beheim, Dan Cummings, Suhail Ghafoor, Paul L. Hooper, Daniel Eid Rodriguez, Andrei Irimia, Margaret Gatz, Wendy Mack, Chris Rowan, Michael Miyamoto, Kenneth Buetow, Caleb Finch, L. Samuel Wann, Adel Allam, Randall C. Thompson, Gregory Thomas, Hillard Kaplan, Jonathan Stieglitz, Benjamin Trumble, Michael D. Gurven

**Affiliations:** ^1^ Institute of Behavioral Science, University of Colorado, Boulder, Boulder, CO 80309, USA.; ^2^ BirthRites Lise Meitner Research Group, Max Planck Institute for Evolutionary Anthropology, 04103 Leipzig, Germany.; ^3^ Department of Anthropology, Pennsylvania State University, State College, PA 16802, USA.; ^4^ MemorialCare Health Systems, Fountain Valley, CA, 92708, USA.; ^5^ Department of Anthropology, University of Utah, Salt Lake City, UT 84112, USA.; ^6^ Department of Human Behavior, Ecology, and Culture, Max Planck Institute for Evolutionary Anthropology, 04310 Leipzig, Germany.; ^7^ Economic Science Institute, Chapman University, Orange, CA 92869, USA.; ^8^ Center for Evolution and Medicine, Arizona State University, Tempe, AZ 85287, USA.; ^9^ Department of Anthropology, University of New Mexico, Albuquerque, NM 87131, USA.; ^10^ Department of Medicine, Universidad de San Simón, Cochabamba, Bolivia.; ^11^ Leonard Davis School of Gerontology, University of Southern California, Los Angeles, CA 90089, USA.; ^12^ Departments of Quantitative and Computational Biology and Biomedical Engineering, University of Southern California, Los Angeles, CA 90089, USA.; ^13^ Centre for Healthy Brain Aging, Institute of Psychiatry, Psychology and Neuroscience, King’s College London, Strand London WC2R 2LS UK.; ^14^ Center for Economic and Social Research, University of Southern California, Los Angeles, CA 90089, USA.; ^15^ Keck School of Medicine, University of Southern California, Los Angeles, CA 90089, USA.; ^16^ School of Medicine, University of Nevada, Reno, Reno, NV 89557, USA.; ^17^ Rowan Cardiology, Reno, NV 89557, USA.; ^18^ Division of Cardiology, Mission Heritage Medical Group, Providence Health, Mission Viejo, CA 92691, USA.; ^19^ Division of Cardiology, University of New Mexico, Santa Fe, NM 87505, USA.; ^20^ Al Azhar University, Cairo, 11651, Egypt.; ^21^ Saint Luke’s Mid America Heart Institute, University of Missouri–Kansas City, Kansas City, MO 64111, USA.; ^22^ MemorialCare Heart and Vascular Institute, Fountain Valley, CA 92708, USA.; ^23^ Institute for Advanced Study in Toulouse, 31080 Toulouse Cedex 06, France.; ^24^ Institute of Human Origins, Arizona State University, Tempe, AZ 85287, USA.; ^25^ School of Human Evolution and Social Change, Arizona State University, Tempe, AZ 85287, USA.; ^26^ Department of Anthropology, University of California Santa Barbara, Santa Barbara, CA 93106, USA.

## Abstract

Porous cranial lesions (cribra cranii and cribra orbitalia) are widely used by archaeologists as skeletal markers of poor child health. However, their use has not been validated with systematic data from contemporary populations, where there has been little evidence of these lesions or their health relevance. Using 375 in vivo computed tomography scans from a cohort-representative sample of adults aged 40+ years from the Bolivian Amazon, among food-limited, high-mortality forager-farmers, we identified cribra cranii on 46 (12.3%) and cribra orbitalia on 23 (6%). Cribra orbitalia was associated with several hallmarks of compromised immune function, including fewer B cells, fewer naïve CD4^+^ T cells, a lower CD4^+^/CD8^+^ T cell ratio, and higher tuberculosis risk. However, neither lesion type predicted other physician-diagnosed respiratory diseases, other markers of cell-mediated immunity, or hemoglobin values. While cribra orbitalia shows promise as a skeletal indicator of health challenges, our findings do not support the continued practice of using these lesions to infer anemia in adults.

## Teaser

Skeletal indicators of childhood stress are associated with cellular indicators of compromised immune function in adults.

## INTRODUCTION

Studies of population health in the archeological literature—including the health impacts of shifts in subsistence and social structure such as those accompanying the agricultural revolution—rely largely on evidence from human skeletal remains. The pathological skeletal features most often reported in these studies are common, nonspecific indicators of physiological stress that develop early in life and can remain visible throughout adulthood: namely, dental enamel defects, stunted growth, and porous lesions of the cranial bones (*1*–*4*). All three have been interpreted as evidence of poor health in past populations, attributed to chronic systemic infections, heavy parasite loads, food insecurity, or inadequate dietary diversity (*5*–*8*). However, whereas enamel defects and growth stunting are routinely assessed in contemporary health care settings (*9*, *10*), porous cranial lesions (PCLs) are rarely reported outside of archaeological contexts, with only a subset of lesion morphology radiographically recognized in clinical settings (*11*). Despite their prominent role in building narratives of past population health, the health implications of PCLs have not been well explored in population-representative samples of living people. Given this disparity, either archaeologists are overinterpreting skeletal lesions with little true relevance to health, or clinical medicine is overlooking a risk marker of lifelong health challenges.

### The interpretation of PCLs

PCLs are characterized in dry bone by the presence of numerous small holes, typically 1 mm or less in diameter, penetrating the normally smooth outer surface of the bones of either the orbital roofs [termed cribra orbitalia (CO)] or the cranial vault [termed cribra cranii (CC) or porotic hyperostosis if accompanied by expansion of the cranial marrow space], where porosity is typically found symmetrically distributed adjacent to the lambdoid or sagittal cranial sutures (*5*, *12*). While researchers in the mid-20th century considered orbital and vault porosity both to be manifestations of severe, infant-onset anemia (*13*–*15*), evidence has accumulated in recent decades that porous lesions of the orbital roofs and cranial vault share a range of overlapping but not identical causes that are likely not yet fully specified (*7*, *16*). Current bioarchaeological consensus considers PCLs in either area of the cranium to be nonspecific skeletal indicators of physiological stress because their known or suspected causes include a range of nutritional deficiencies, infections, and trauma, although anemia is invariably the first diagnostic possibility to be considered (*17*). Regardless of their specific causes, PCLs are believed to form predominantly in the first, and possibly second, decade of life because archaeologists report lesions without evidence of partial healing almost exclusively in the skeletal remains of individuals younger than 15 (*18*–*21*). The partially healed lesions observed in adults are thus predominantly general indicators of childhood medical history. The existence of unobservable, fully healed lesions is theorized (*22*), but in the absence of longitudinal clinical data on lesion morphology, the rate at which these lesions might heal to the point of invisibility in surviving individuals is unknown.

### Bioarchaeology and the developmental origins of health and disease

In archaeological contexts, PCLs—particularly CO— are more common in children than adults and more common in individuals in their twenties and thirties than they are in older adults (*20*, *22*–*27*). This pattern is typically interpreted as evidence of lesion-associated mortality risk that extends well beyond the period of lesion formation and fits within the framework of the developmental origins of health and disease (DOHaD), which aims to link early-life stressors with health risks in adulthood (*27*). While DOHaD studies in contemporary populations have focused primarily on links between early-life cues of environmental quality and subsequent risk of noncommunicable diseases in older adults (*28*), archaeological studies that invoke the DOHaD paradigm are unavoidably constrained to consider the relationship between skeletal indicators of early-life physiological disruption and the relative risk of death, rather than disease, at subsequent ages (*27*). Heightened risk of disease is inferred from heightened risk of death. The current study is able to test this assumed relationship by looking directly at associations between skeletal indicators of early-life experience (PCLs) and downstream measures of health in living adults.

**Fig. 1.**
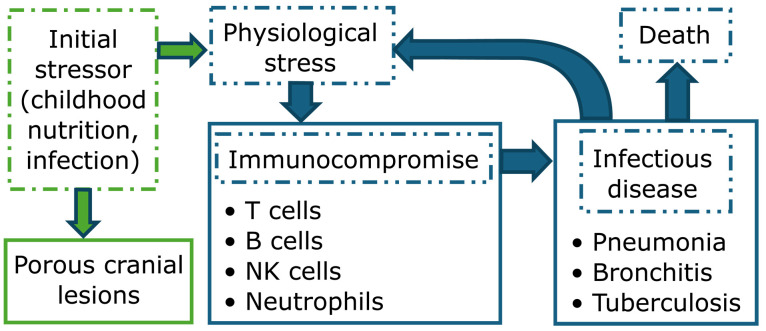
Conceptual framework. Causal pathways through which PCLs may be associated with health outcomes among older adults. Dashed boxes indicate variables not measured directly in the current study. Green indicates processes that are confined to childhood, while items in blue are relevant across an individual’s entire life span. NK cells, natural killer cells.

Where skeletal indicators of childhood stress predict higher mortality risk in childhood and young adulthood, the pathway for this relationship is presumably through prolonged, heightened susceptibility to the primary causes of death among these age groups in past environments (Fig. 1). Respiratory infections fit this description: They are consistently a leading cause of death in historic records (*29*, *30*) and low-income groups, including those still engaged in traditional means of subsistence such as hunting and gathering (*31*), and initial infection appears to have far-reaching effects on subsequent morbidity (*32*). Respiratory infections may therefore be a plausible mediator between exposure to lesion-causing stressors and subsequent premature mortality. Recent studies of contemporary and historic pediatric mortality samples report that the orbital lesions of CO are significantly more common in children and adolescents with fatal respiratory infections (*33*, *34*). However, it has not yet been investigated whether PCLs predict respiratory infections among adults, or whether these lesions have lasting implications for compromised adult health more broadly.

### Strengths of the current study

To test whether these apparent skeletal indicators of childhood stress are associated with distinct health trajectories, including heightened risk of respiratory infection at older ages, we leverage existing cranial computed tomography (CT) scans and cross-sectional and longitudinal health data collected over 16 years from a cohort-representative sample of living adults in a food-limited, high-infection environment. The Tsimane population examined here has a high prevalence of respiratory infections, parasitic infection, and childhood anemia—all of which have been correlated with PCLs in radiographic studies (*33*, *35*, *36*), making both the presence and the absence of PCLs in this population a relevant finding for archaeological interpretations of population health.

The current study has several strengths relative to past research on PCLs in the clinical and archaeological sciences. First, the surface porosity that defines these lesions for the paleopathologist has not been radiologically visible until recently, with the development of multislice CT scanners that enable volume-rendered images of surface features with submillimeter resolution and bridge the gap between archaeological and clinical visualization of the skeleton. Second, the use of a living cohort bypasses some limitations of archaeological contexts which, being limited to life experiences and illnesses that manifest in the skeleton, are poorly equipped to test the relationship between skeletal lesions and experiences of morbidity. The health implications of skeletal lesions without well-established interpretations based on clinical findings are more difficult to discern from archaeological skeletal series, making bioarchaeology’s well-known equifinality problem, the osteological paradox (*37*), more relevant in analyses of lesions like PCLs. Moreover, because the study sample is composed of those who survived into and beyond their fifth decade, patterns of morbidity in older Tsimane adults are more easily applied to archaeological inference than patterns of morbidity in living children would be. Children in the archaeological record are only ever children who did not survive their childhoods. Consequently, older individuals in a mortality sample are more representative of health among their living peers than are the youngest nonsurvivors in a mortality sample.

### Study population

The indigenous Tsimane population (population ≈ 17,000) who reside in the Beni department of Amazonian Bolivia live a mostly subsistence lifestyle in a tropical environment (*38*), precisely the setting in which the archaeological literature reports a high prevalence of PCLs (*39*). Most Tsimane villages have little public infrastructure, minimal access to electricity, and limited access to modern medical resources (*40*). The population experiences high mortality, with death rates across the life course similar to European mortality from the 1800s (*41*). Respiratory infection is the most commonly documented category of illness and has been documented as the leading cause of death for Tsimane of all ages (*42*). Starchy horticultural staples (62% of calories), wild game (6% of all calories), and fish (16%) make up most of the Tsimane diet, although consumption of cooking oil and other shelf-stable, market-purchased foods (recently estimated at 8% of average daily calories per person) is increasing (*43*). Inflammation due to infection is prevalent: Total white blood cell counts among the Tsimane are roughly 1.5 times higher than the US average (*44*). Hookworm and other parasitic worms are endemic to the Tsimane territory, and 89% of Tsimane have eosinophilia (*44*), an investment in immune responses to these parasites.

Childhood anemia is often cited as a probable cause of PCLs, particularly anemia due to blood loss from intestinal parasites (*5*), and anemia is widespread among Tsimane children despite high intake of dietary iron (*43*). Hookworm infection is common (*34*, *45*, *46*), suggesting hookworm-driven blood loss as a major cause of anemia. However, anemia should not automatically be considered the underlying cause of PCLs in this population. Most Tsimane children with hemoglobin values below the threshold that defines anemia do not have the iron deficiency (*47*) that characterizes parasite-driven anemia. Anemia of inflammation is a stronger candidate for many cases of anemia in this population, and the potential for anemia of inflammation to cause PCLs has not been clinically documented.

### Study goals

**Fig. 2.**
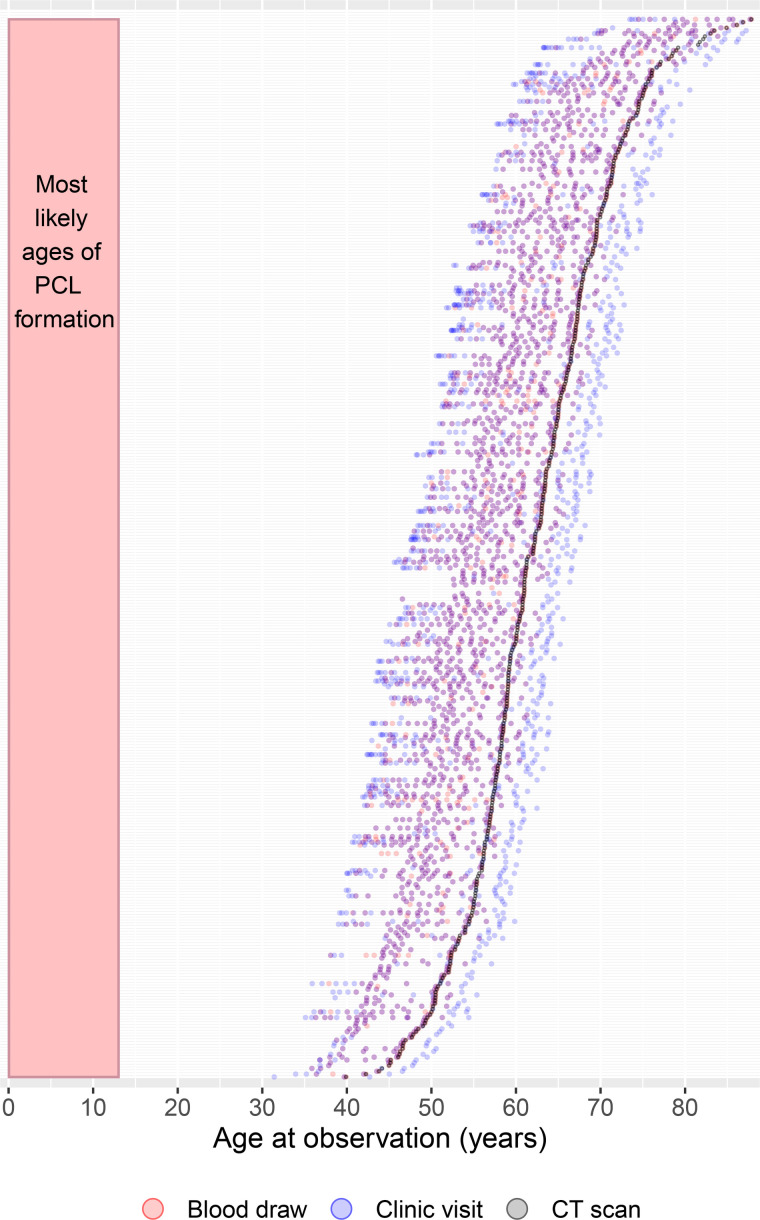
Timing and frequency of available health data. Ages at time of data collection (points) for each of the 375 individuals in the study relative to the inferred age range of potential PCL development (pink rectangle) and the age at which each person’s PCL status is observed from their cranial CT scan (grey points). Each person in the study is plotted at a unique position on the *y* axis, resulting in horizontal data timelines for each individual.

The current study asks: If PCLs, which most likely formed in the first decade of life, are still observable in Tsimane adults now living in their fifth decade and beyond, do these adults with PCLs have different health profiles from their peers without visible PCLs? It describes the demographic profile of older Tsimane adults with PCLs and investigates the health correlates of these skeletal lesions using mixed longitudinal data on a range of health measures (Fig. 2)—clinical diagnoses, leukocyte subtype counts, and hemoglobin—collected during routine physical exams by Tsimane Health and Life History Project (THLHP) physicians who visit participating villages on a roughly annual basis (*44*).

1) Are PCLs associated with respiratory infections in older adults?

We first focus on pulmonary tuberculosis and other respiratory infections both because of the association between PCLs—most notably the orbital lesions, CO—and fatal respiratory infection reported in recent publications (*33*, *34*) and because of their high prevalence, high morbidity burden, and notable contribution to mortality across the life course in the Tsimane and other populations (*38*, *42*). We consider PCL presence as a predictor of the incidence of clinical respiratory diagnoses of infectious origin. Incidence of upper respiratory infections is modeled separately from lower respiratory infections because lower respiratory infections tend to last longer and can be more severe than upper respiratory infections (*48*). The impact of CO and CC presence on the hazard of receiving a tuberculosis diagnosis is analyzed separately from other respiratory conditions because tuberculosis in adults is often a reactivation of a latent infection acquired earlier in life (*49*) rather than a short-term illness with acute onset following exposure.

2) Are PCLs associated with differences in adult immune cell populations?

To explore a possible relationship between lesion-causing experiences in childhood and adult immune function, we also investigate whether CO and CC are associated with differences in adult immune cell populations. Models of the association between cranial lesion status and total leukocyte counts, lymphocyte counts, and granulocyte counts are primarily exploratory. However, for the subset of 196 individuals with flow cytometry measures, the ratio of CD4 to CD8 T cells is expected to be negatively associated with the presence of PCLs. The CD4/CD8 ratio is a measure of immune competence that has been validated as an integrative marker of biological age (*50*, *51*). A low CD4/CD8 ratio is considered an immune risk phenotype and an indicator of immune senescence (*51*). Ratio values below 1 have been associated with higher mortality risk and higher measures of oxidative stress among older adults (*52*, *53*).

3) Are PCLs associated with anemia in adults?

Last, this study examines the long-held interpretation of PCLs as skeletal indicators of anemia. Even if one were to assume that childhood anemia is the primary cause of PCLs in this population, having had anemia in childhood is not expected to be a strong predictor of having anemia at least four or more decades later, except in cases of hereditary anemias, which have not been documented in the Tsimane population. Consequently, despite the widespread rote attribution of PCLs to anemia, we do not expect hemoglobin values to show any correlation with PCLs among the adults in this study.

Although the causes and physiology of these skeletal lesions are ongoing areas of research, this study relies on previously collected data that are not suited to identifying specific early-life causes of PCLs in the study population. The scope of the current study focuses specifically on the health profiles of adult individuals for whom we have both a high-resolution cranial CT scan and associated biomedical data from the past two decades.

## RESULTS

### Descriptive statistics

**Fig. 3.**
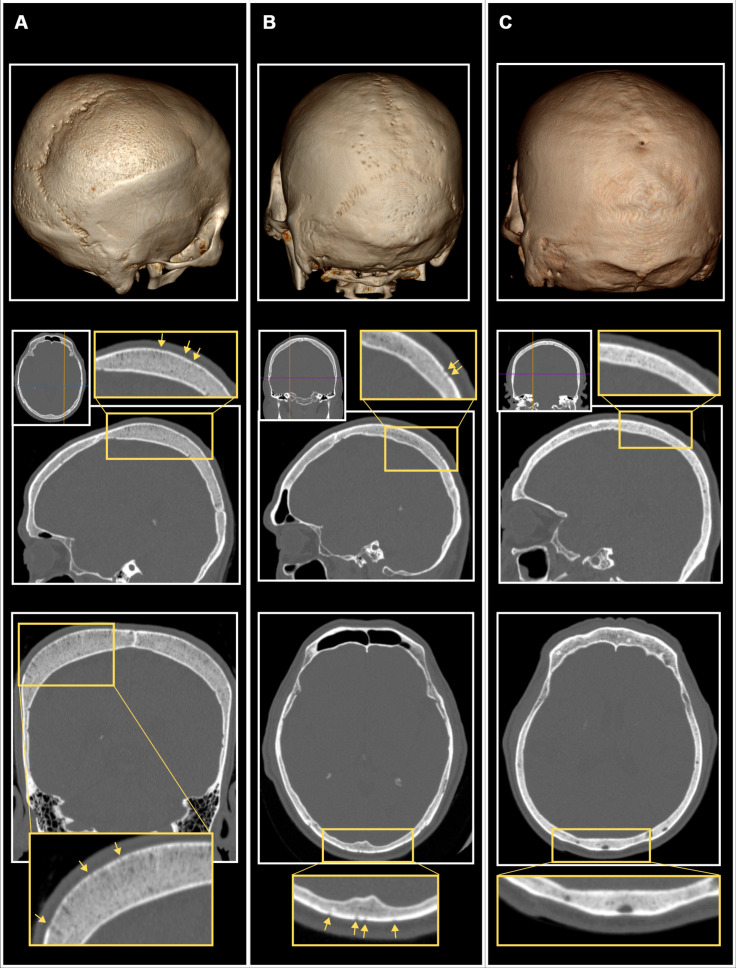
CT appearance of cranial vault lesion (CC) presence and absence in study participants. (**A**) Ectocranial porosity with accompanying marrow expansion and hair-on-end appearance of cranial diploe. (**B**) Ectocranial porosity on the posterior parietal bones and occipital squama. (**C**) Absence of CC. Arrows indicate individual foramina.

**Fig. 4.**
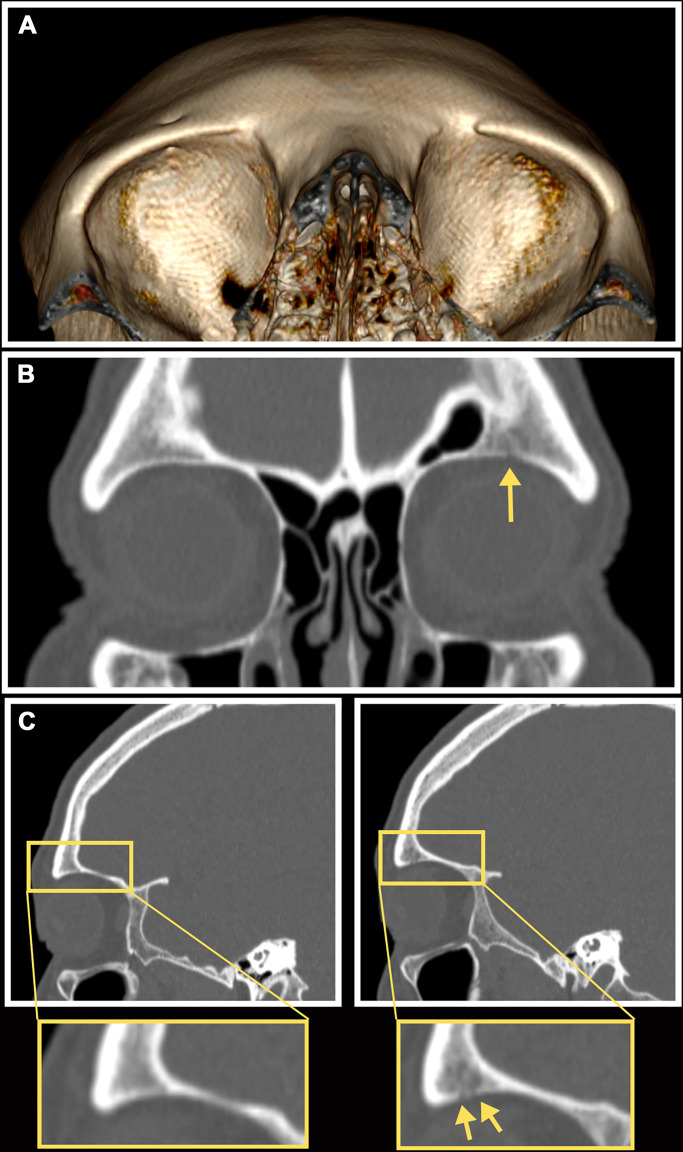
CT appearance of CO presence and absence in study participant. (**A**) Volume-rendered CT reconstruction showing an individual with CO present in the left orbit and recorded as absent in the right orbit. (**B**) Coronal slice of CT MPR showing the same individual. (**C**) Left: Sagittal slice of right orbit showing intact orbital roof. Right: Sagittal slice of left orbit with visible porosity in the anterior orbital roof. Arrows indicate individual foramina.

Applying a recently validated protocol for evaluating PCLs using CT scans (see Materials and Methods) (*54*), evidence of CO and CC was evaluated in scans for 375 living adults aged 40+ years from the Tsimane. Of the 375 cranial CT scans, scanning artifacts prevented the evaluation of CC in two individuals (*n* = 373) and CO (*n* = 374) in one person. Of the 372 cranial scans that could thus be evaluated for both CO and CC, PCLs were observed on 64 (17.2%). Forty-six (12.3%) of the 373 individuals with observable cranial vaults had visible pitting or porosity of the cranial vault consistent with CC (Table 1), most frequently on the occipital squama. Only a single case of vault porosity was true porotic hyperostosis, accompanied by visible expansion of the cranial marrow space and also exhibiting hair-on-end sign (Fig. 3). CO was visible in 23 of 374 scans with observable orbits (6.1%) (Fig. 4 and fig. S2). CO was conservatively considered absent in a further 37 individuals who presented with ambiguous evidence of CO (fig. S2); in sensitivity analyses, considering these to be positive cases of CO did not change regression model results (described below). CO and CC were not significantly associated with each other (chi-square = 1.17, *P* = 0.28); only five individuals had concurrent orbital and vault porosity. Because of the visibility limits imposed by the scans’ resolution (pixels of 0.39 mm^2^ in axial plane images and 0.39 mm × 0.675 mm in the sagittal and coronal planes) and our conservative approach to identifying lesions (see Materials and Methods), the vault and orbital lesion frequencies reported here should be interpreted as the minimum prevalence of PCLs in older Tsimane adults.

**Table 1.** Frequency of PCLs in Tsimane adults, by sex. The total number of observable crania differs for CO (*n* = 374) and CC (*n* = 373).

Sex	CO (*n*)	CO (%)	CC (*n*)	CC (%)	Mean age (SD)
**Female**	12	7.23	11	6.67	62.22 (8.2)
**Male**	11	5.29	35	16.83	62.72 (8.65)
**All**	23	6.15	46	12.33	62.5 (8.45)

### Demographic patterns of PCLs in Tsimane adults

**Fig. 5.**
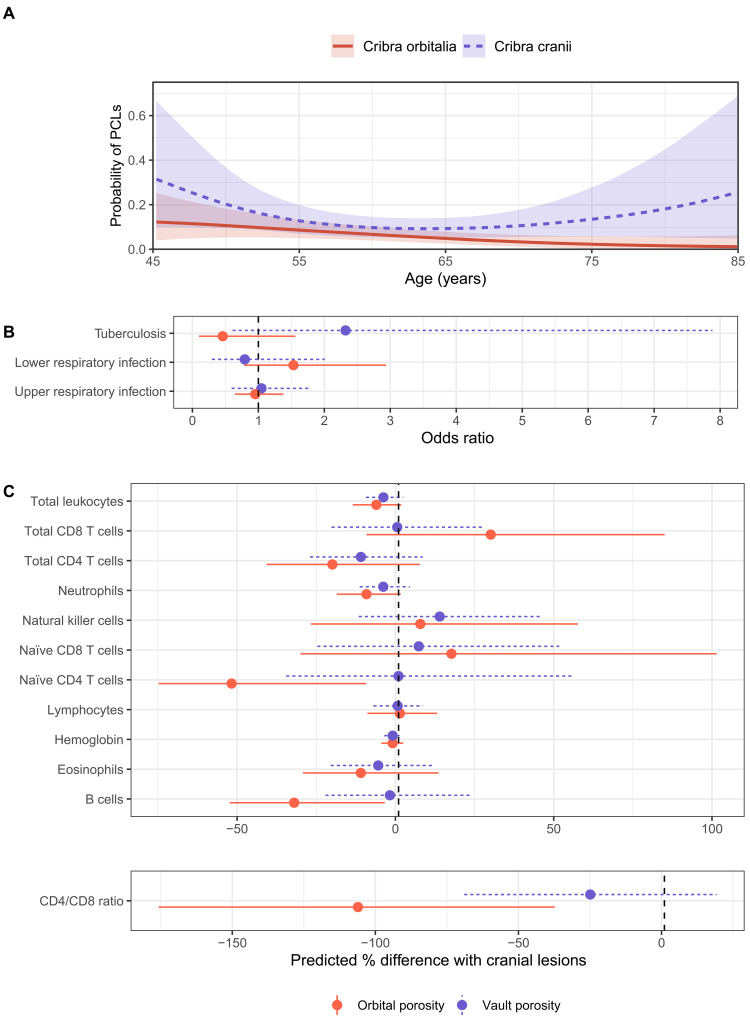
Associations between PCLs, age, diagnoses, and biomarkers. (**A**) Probability of having CO (red solid line) is negatively associated with age but probability of having CC (blue dotted line) is not. Plot shows predicted probability of lesion presence at each age from a univariate, nonlinear logistic regression with a thin plate regression spline. (**B**) Estimated OR (and its 95% CrI) for the effect of each lesion on age-adjusted risk of a respiratory diagnosis. Bayesian multilevel logistic models for lower and upper respiratory infection (*n* = 2886 clinic visits) include a random effect for individual participant, while logistic models for tuberculosis evaluate the association of PCLs with an individual’s probability of ever receiving a TB diagnosis. (**C**) Percent difference in predicted values from multilevel linear models for each biomarker when lesions are present rather than absent, calculated for a female of the median sample age. A value of −100 on the *x* axis therefore means a biomarker’s predicted value in the presence of a lesion is one half of the value predicted for a female of the same age without a lesion. See the Supplementary Materials for the number of individuals and observations for each biomarker.

Study participants ranged in age from 39 to 87 years old. In a Bayesian logistic regression modeling age as a linear term, CO was negatively associated with age [odds ratio (OR) = 0.93 [95% credible interval (CrI): 0.88 to 0.99] per additional year] (Fig. 5A and fig. S2). This result remains whether ambiguous orbital lesion cases are considered present, absent, or excluded entirely from the models and when considering lesion cases involving one or both orbits. CC was more often observed in males [OR = 2.86 (95% CrI: 1.45 to 6.09); Table 1 and table S1]. The relationship between CC and age, however, was difficult to estimate due to small sample sizes at the extremes of the participant age range (Fig. 5A). CC did not show a significant linear relationship with age [OR = 0.99 (0.95, 1.02) per additional year].

### PCLs and respiratory infection risk

Associations of PCLs with respiratory infections were evaluated in Bayesian multilevel logistic regression models that included 2886 study visits among 372 participants over 20 years. Neither type of lesion showed a strong association with incidence of upper or lower respiratory infections (table S2). Upper respiratory infections were diagnosed at 11.5% and lower respiratory infections at 8.6% of medical visits. The age-adjusted estimate of risk of lower respiratory infection was slightly lower in the presence of CO (OR = 0.79, 95% CrI: 0.30 to 2.0) and slightly higher in the presence of CC (OR = 1.24, 95% CrI: 0.66 to 2.30) but with wide uncertainty on the direction of those associations. Age-adjusted estimates of upper respiratory infection risk were similar in the presence and absence of lesions (Fig. 5B). We also tested whether CO could be attributed to localized eye infections in adulthood but found no association of CO with eye infections (table S3).

Overall, 39 (10%) of the 372 individuals with observable status for both types of PCLs presented with symptomatic tuberculosis (TB) at a clinic visit during the 20-year period of observation (2002–2022). A Bayesian logistic regression adjusting for age at first TB diagnosis (for TB^+^ individuals) or age at most recent medical visit found that CO was associated with an estimated 2.4 times the risk of ever being diagnosed with tuberculosis but with wide uncertainty on that estimate (95% CrI: 0.61 to 7.70). The estimated association of CC with tuberculosis (OR = 0.46, 95% CrI: 0.11 to 1.54) had a narrower range of uncertainty but, like CO, did not reach the threshold for statistical significance. Given that only three tuberculosis cases presented with CC and four with CO (table S4), the direction of these effects should be interpreted with caution.

### PCLs and adaptive immunity

Associations of PCLs with measures of adaptive immunity and hemoglobin were evaluated using multilevel linear models with log transformation of immune cell populations. Among the subset of individuals with flow cytometry measures (252 visit measures among *n* = 196 participants), those with CO present (*n* = 7; 10 visit measures) had a B cell count that was 30.7% lower [95% CrI = (−51.6, −1.4)], as well as lower CD4^+^ T cell counts and higher CD8^+^ T cell counts that together yield a CD4/CD8 ratio 0.49, or 100.7%, lower [95% CrI = (−170.8%, −33.5%)], compared to their peers without lesions (Fig. 5C). Low total CD4^+^ counts were largely driven by lower naïve CD4^+^ populations which were 49% lower [95% CrI = (−73.8, −2.4)] for those with CO. Unlike CO, CC was not associated with systematic differences in white blood cell subtypes (Fig. 5C and table S5). Adjusting for current infection did not alter results associated with either lesion type (table S6).

### No associations with adult anemia

Hemoglobin data were collected every 1.6 years on average (range: 1 to 15 years), with an average of six (range: 1 to 13) measurements per person, including hemoglobin on the date of CT scanning. Neither CO nor CC was associated with differences in hemoglobin among Tsimane adults (Fig. 5C and tables S5 and S7) or higher probability of anemia (table S7). Even the single individual whose cranial vault lesions presented with marrow expansion and hair-on-end appearance—considered pathognomonic for severe childhood anemia—had normal adult hemoglobin values.

## DISCUSSION

We found that the PCL CO was negatively associated with age in Tsimane adults (aged 40+ years). This is consistent with the age patterning that would result from a higher rate of adult mortality among individuals with CO, but the as-yet-unknown pace of remodeling for these skeletal lesions could also contribute to CO’s negative relationship with age. CO also predicted lower age-adjusted counts of B cells and naïve CD4^+^ T cells and a lower CD4/CD8 T cell ratio, an immune profile that has been associated with vulnerability to infection in other populations (*55*). CO’s connection to diagnosed health conditions was less clear. There was weak evidence (based on small numbers of infected persons) that CO was associated with symptomatic tuberculosis (OR = 2.4, 95% CrI: 0.61 to 7.70), and CO was not associated with the presence of upper respiratory infections. CC, by contrast, had no clear association with age or any of the health metrics in this study but was more commonly observed in males. Last, neither lesion showed any relationship to adult anemia.

The study results establish that these archaeologically common cranial lesions are also present with moderate to high prevalence in this contemporary population, despite the broad absence of evidence for these lesions in clinical settings. The lesion frequencies reported in the current study should be considered estimates of minimum lesion prevalence in Tsimane adults, considering the lower resolution of CT imaging compared to the sensitivity of direct visual examination of archaeological remains. Despite this conservative threshold for detection, Tsimane lesion prevalence is within the range of archaeological findings (*25*).

The negative association between CO and age in Tsimane adults is consistent with a pattern of higher adult mortality risk for those who developed CO in childhood. If PCLs had no relationship to mortality risk—and never healed completely—then we would expect lesion frequency to be uniform across adult ages. The demographic patterning of PCLs in Tsimane adults is also consistent with bioarchaeological studies, which often report a weaker negative relationship between age and CC than age and CO (*56*–*58*). Alternatively, fewer visible lesions at older ages could be caused by complete healing of these lesions in some adults. The pace of lesion healing is still unknown; however, the presence of these childhood lesions in older adults suggests that the pace of such healing is either highly variable or very slow. The existence of unobservable, fully healed lesions remains a theoretical confounding factor in interpretations of the age profile of PCLs across the life course.

Although CC was almost three times more common in males than females in adults aged 40+ years, it is unclear how to interpret that difference without knowing the initial sex differences in the frequency of this childhood skeletal lesion. PCL frequency is not consistently sex biased among adults in archaeological studies. Given that PCLs form predominantly in childhood, adult sex differences may be the product of sex-specific risks during childhood (*59*). Although a few exceptional contexts with known sex of nonadults report more PCLs in males than in females (*19*, *60*), most archaeological studies do not perform the necessary chemical analyses to determine sex from the skeletal remains of prepubertal individuals, leaving the sex-differentiated risks of PCLs over the life course an open topic for future study.

The immune cell correlates of CO in the current study support the idea that individuals who experience lesion-causing stress in early childhood also experience elevated morbidity across the life course—lower CD4/CD8 T cell ratio, fewer naïve CD4 cells, and fewer B cells have been shown to develop as a consequence of chronic immune activation (*51*, *53*, *61*). CD4/CD8 ratio values below 1 are clinically considered an immune risk phenotype (*51*). A total of 40% of CD4/CD8 ratios in individuals with CO fell below this threshold, compared to 19% of those without CO. Although immune activation is a well-described and widespread aspect of Tsimane life experience at all ages, the distribution of CD4/CD8 ratios in Tsimane individuals is similar to German and English references in all but the 50+ age group, where Tsimane values are significantly lower (*44*, *62*, *63*). Low naïve CD4^+^ counts among older Tsimane adults with CO suggests that these individuals may have a depleted capacity to mount immune responses to previously unencountered infections and points to influence of childhood physiological stress on trajectories of disease experience. If lesion-causing processes have long-term costs to physiological function—or if lesions are more likely to form in individuals born more vulnerable to environmental stressors—then the development of PCLs might be a harbinger of lifelong vulnerability to morbidity and mortality.

Archaeological evidence suggests that the mortality risks associated with PCLs are concentrated in childhood (*23*), but results of the current study suggest that the mortality differential associated with CO continues well into older adulthood. The adults in the current study, aged 40 + years, fall into an age range for which skeletal estimates of age are notoriously imprecise, leading to a tendency for archaeological studies to lump all individuals estimated to be older than 50 or 60 years into a single category against which lesion frequencies in younger age categories can be compared (*27*). As a result, the age patterning of lesion frequency patterns among older adults has not been well explored, and the lifelong consequences of lesion-causing stress have been underidentified.

The current study provides a very conservative estimate of the health costs associated with PCLs in the Tsimane population. Although cohort representative, our sample is composed of adults who survived into their fifth decade and beyond, and the cranial lesion status of cohort members who did not survive to these ages is unknown. Tsimane people experience high infant and childhood mortality; nearly 25% of the population does not survive childhood. A sample of older adults may comprise a robust subset of survivors for whom lesion-causing stress has little measurable impact on lifelong health experience, even if PCLs are associated with higher population-level morbidity and mortality at younger ages. Since the morbidity and mortality risk attributed to cranial lesions in archaeological settings is concentrated among children and young adults, the current study’s focus on older adults likely underestimates the lifetime impact of lesion-causing processes in the study population.

Last, our results do not support the continued use of PCLs as skeletal indicators of adult-onset anemia, [e.g. (*64*, *65*)]. Regardless of whether anemia is the primary cause of childhood PCL formation in this population, PCLs are not informative about anemia status in Tsimane adults. Anemia has long been invoked as a predominant cause of PCLs, and given that anemia is widespread in Tsimane children (*43*), we cannot rule out anemia as a cause of the PCLs observed in Tsimane adults. However, the physiology of bone marrow changes with age in ways that render adult-onset PCLs a highly unlikely consequence of adult-onset anemia (*66*–*68*). With almost a decade of semiannual hemoglobin measures on study participants, we found that hemoglobin values were similar for individuals with and without PCLs. We recommend researchers exercise care to interpret these skeletal lesions in ways that are consistent with the current state of knowledge on skeletal physiology.

### Current considerations and future opportunities

The current study was able to investigate assumptions and outstanding questions in human skeletal pathology from a new vantage point, directly examining the relationship between PCLs and later life morbidity in a variety of health domains. Nevertheless, the limits of the available data must be acknowledged. First, clinical CT scans cannot distinguish between PCLs that are unremodeled, indicating active lesion formation, and PCLs with rounded margins of individual foramina, indicating lesion healing. As a result, the specific age of lesion formation or duration of lesion-causing processes cannot be inferred from a single CT observation per individual. Second, only lesions composed of porosity with a diameter above the scan resolution are observable from THLHP cranial scans. If finer porosity is also associated with health outcomes, then these unobservable cases may obscure the true extent of the health correlates of PCLs in this sample, making the current study a conservative test of this relationship. Likewise, clinical diagnostic data provided information on incidence of respiratory infections but lacked measures of severity or duration for individual bouts of infection, which are themselves salient contributors to overall morbidity burden and may be more relevant measures of morbidity-related mortality risk than mere presence.

Last, age-related thinning of the orbital roofs may contribute to the lower frequency of CO observed at older ages. Thinning of the orbital roof is a known change in cranial morphology associated with aging, and regions of the skull that are substantially thinner than the scans’ slice width (here, 0.625 mm) are not captured well by CT. As a result, it may be more difficult to visualize orbital lesions in older individuals. The limitations of CT for positively identifying cases of CO (*53*) may be exacerbated by age-related thinning of the orbital roofs, although given that the age range of individuals with ambiguous cases of CO was similar to those with clearly visible cases (fig. S3), this seems unlikely to be responsible for CO’s negative association with age.

CO, a skeletal lesion that develops in early childhood and is largely ignored by clinical practice but ranks among the most common pathological indicators in archaeological studies of human skeletal remains, is associated with evidence of persistent immune activation among adults in a contemporary subsistence population. These findings address the inferential foundations of bioarchaeological studies of health, which have relied on these lesions as evidence of individual health insults without being clear about the assumptions on which these interpretations rest. The inescapable confounding factors inherent to reconstructing population health from the dead, collectively known as the osteological paradox (*37*), are magnified when the relationship between skeletal features and disease experiences are unknown among the living, as is the case for PCLs. This study identifies these skeletal lesions as possible risk markers for poor health in contemporary populations. These lesions may merit more attention in clinical practice, at least as incidental findings on radiological exams. Future studies of individuals from a wide range of ages and backgrounds are needed to determine whether PCLs consistently predict morbidity and premature mortality across populations and across the life course. The current study is an initial stride toward an integrated approach to the study of health in populations past and present and a promising first demonstration of skeletal indicators of childhood stress as a window into the lifelong effects of childhood health insults in prehistory.

## MATERIALS AND METHODS

### Study sample

Data for this study come from the THLHP (https://tsimane.anth.ucsb.edu/), a longitudinal study of health and aging among Tsimane communities that has been running since 2002 (*38*). Cranial CT scans were obtained from a cohort-representative sample of Tsimane adults over age 40 [see (*64*) for details of the sampling strategy]. All Tsimane individuals with a cranial CT scan obtained in 2017 or 2018 were observed during clinical medical exams with blood sampling between 2004 and 2020 (fig. S4 and table S8). Clinical and health history data were collected by the THLHP mobile medical team during annual visits following routine medical examinations (patient history, symptom investigation and clinical diagnoses, blood pressure and temperature, and height and weight). THLHP collection protocols for biomedical data are outlined in detail in numerous publications (*44*, *69*–*74*).

### Ethics approval

The study was reviewed and approved by the Institutional Review Boards (IRBs) of the University of California-Santa Barbara (#3-19-0908), the University of New Mexico (#07-157), and Universidad Mayor San Simon, Cochabomba Bolivia. The Gran Consejo Tsimane, the governing body overseeing Tsimane affairs and research projects, additionally reviewed and approved the studies from which Tsimane data are drawn. Informed consent was obtained at both the community and individual participant levels. During a community meeting open to all residents, communities determined collectively whether the study would be conducted. To date, all communities that have been approached have approved the study. Individuals gave informed consent before each medical visit, procedure, and biospecimen collection.

### Medical diagnoses

Medical diagnoses were determined on-site during routine physical exams by physicians with the THLHP mobile medical team, using diagnostic protocols based on symptomatic presentation and supported by white blood cell differentials and, in some tuberculosis cases, sputum tests (see the Supplementary Materials for diagnostic protocols). The 375 individuals in the current study totaled 2886 visits with the medical team between 2004 and 2020 (median number of visits = 7; range = 1 to 20; median time range across all community visits = 12 years). For the analysis of respiratory infection incidence, bronchitis and pneumonia were classified as lower respiratory tract infections; upper respiratory infections included pharyngitis, acute nasopharyngitis, sinusitis, and tonsillitis. Respiratory conditions likely directly related to chronic smoke exposure (e.g., pulmonary fibrosis) or of entirely noninfectious origin (e.g., nasal polyps) were excluded.

### Biomarker collection

Five-part blood differentials were collected for all individuals at all medical exams. Venous blood samples were collected by certified Bolivian biochemists into a heparin-coated vacutainer. Total white blood cells and hemoglobin values were measured using a QBC Autoread Plus dry hematology system (QBC Diagnostics) directly after blood draw, and leukocyte subtypes were counted manually using microscopy and a hemocytometer (*44*).

Flow cytometry was measured on all individuals brought to the THLHP clinic in San Borja between late 2011 and 2014. Of these, 196 individuals (unique observations = 252) also had bone-optimized images from cranial scans obtained in 2017–2018 and were thus included in the study. Flow cytometry was conducted on an Accuri C6 Flow Cytometer (BD Accuri Cytometers) within 6 hours of the blood draw in the THLHP clinic in San Borja on fresh heparinized blood. Lymphocytes were labeled with appropriate fluorescently tagged antibodies and categorized as helper T cell (CD4^+^CD8^−^), cytotoxic T cells (CD8^+^CD4^−^), natural killer cells (CD56^+^CD8^−^CD4^−^), and B cells (CD19^+^). Helper and cytotoxic T cells were further classified as naïve (CD45RA^+^) or non-naïve (CD45RA^−^). Absolute counts of each subset were calculated by multiplying the relative percentages determined using a flow cytometer by the total lymphocyte count obtained from the QBC Autoread Plus (*44*).

### CT data collection

CT scans were obtained as part of a THLHP study on cardiovascular health, dementia, and cognitive aging (*75*–*77*). The CT scans used in the current study were collected between 2017 and 2018 and comprise a subset of the 746 scans obtained for a study of Tsimane brain health (*76*); these 375 are the subset whose scans were obtained after the conception of the current study and so included an additional set of images produced using a sharp kernel algorithm for optimizing the visibility of hard tissue. Individuals who consented and were able to travel were transported to the German Busch Hospital in Trinidad, Bolivia for scanning. Cranial CT scans were performed by a licensed radiological technician using a 16-detector row multislice CT (GE Brightspeed, Milwaukee, WI, USA) under the supervision of project clinicians.

Cranial scans were obtained using a slice thickness of 0.625 mm (130 kvp; 140 mAs; pitch, 0.65), and the raw data were processed using a bone-optimized reconstruction algorithm. The first author (A.S.A.) assessed PCL (CC and CO) status on each scan using the freeware DICOM viewer Horos (*78*) following the protocol laid out by Anderson *et al.* (*54*). Briefly, A.S.A. first observed all cross-sectional slices of the scan in coronal, sagittal, or axial planes using multiplanar reconstruction (MPR) with window width and level set to Horos’ bone-optimized preset. MPR images were scored for the presence or absence of three lesion-related traits on the frontal, parietal, and occipital bones: Radial trabecular orientation (radiologic “hair-on-end” sign), superficial porosity of the outer table (ectocranial pitting), and perforation of the outer table (Fig. 3). For orbital roofs, only cortical perforation was noted (Fig. 4). MPR observations of each skull were then corroborated by observing surface features of a rotatable three-dimensional (3D) volume rendering, with color lookup table set to “VR muscle and bone” and the generated light source set to “diffuse.” Surface pitting or porosity was scored as either “fine,” “distinct,” or “trabeculated” for each bone of the cranial vault and each orbital roof, along with degree of observer certainty. All cases were randomized and reassessed 2 weeks later; for the purpose of analysis, only cases for which PCLs were positively identified in both rounds of data collection were considered to have PCLs.

CC was considered present if pitting or porosity composed of more than five distinct foramina was observed on at least one bone of the cranial vault, although porosity composed of distinct individual foramina was only observed on the posterior parietal bones and occipital squama. Because the data for this study were collected by a single observer and the results of previous work (*54*) emphasize that lesions with foramina close to or below the scale of the scan’s resolution (fine or “pinprick” porosity) are difficult for observers to identify accurately, only lesions with larger, distinct foramina were identified as unambiguously present, although any indications of porous changes were noted during data collection to check the robustness of results when marginal lesion cases are included/excluded.

CO was considered present if multiple foramina were unambiguously visible in one or both orbits, viewed in both coronal and sagittal planes of MPR and using 3D volume rendering. Most CO cases identified in the present study fall under Nathan and Haas’ “trabecular type” classification (*79*). CO with the appearance of the “porotic” and “cribrotic” types could not be reliably identified by observers in initial tests (*54*), and false positive cases often took on the appearance of porotic or cribrotic lesions. This study is therefore conservative, limited to observing orbital lesions with more extensive skeletal changes.

### Statistical analysis

All statistical tests used Bayesian regression methods, with default priors from the brms package (*80*) for random effects and weakly informative gaussian priors centered on zero for main effects. Results of each logistic model are reported as an OR with 95% CrI. To model the repeatedly measured health outcomes, multilevel models specified random effects at the participant level. Each regression included either CO or CC presence as a binary variable.

#### Associations of PCLs with age and sex

Demographic differences between individuals with and without PCLs were assessed using logistic regressions with PCL presence (CO or CC) as the dependent outcome variable and age and sex as independent variables. To avoid unwarranted assumptions about the dynamics of the processes that might contribute to age-related patterns in PCL frequency—selective mortality and lesion remodeling—demographic associations were visualized using logistic regressions for lesion presence with a thin plate regression spline for age, grouped by sex. Logistic regressions were also run without a spline, for the ease of directly interpreting model output (table S1); these corroborated results from the spline model were plotted in Fig. 5A.

#### Associations of PCLs with clinical diagnoses

Each diagnosis (upper respiratory infection, lower respiratory infection, and tuberculosis) was treated as a binary outcome variable. Two logistic regression models were run for each diagnosis: one model with CO status as a binary independent variable and one with CC status. All regressions of clinical diagnoses included fixed effects for sex and the individual’s age at the time of the clinic visit. Because study participants were seen at the clinic multiple times over the 20 years of the study period, associations with upper and lower respiratory infection risk were evaluated using Bayesian multilevel logistic regressions with a random effect for individual study participant. In contrast, tuberculosis was modeled as a chronic rather than a recurrent condition; logistic regressions evaluated the association of CO or CC with the probability of being diagnosed with tuberculosis at least once during the study period.

#### Associations of PCLs with leukocytes and hemoglobin

Multilevel linear models of continuous hematological measures included independent variables of sex, age at time of blood draw, and PCL status (either CO or CC) and a random participant-level effect. Leukocyte counts were logged for analysis. Except for the model predicting total leukocyte count, a second set of models added a fixed effect for logged leukocyte count to assess the impact of current infection on associations of PCLs with hematological measures. For hematologic measures of white blood cell subsets, the logged leukocyte covariate was adjusted to exclude the cell subset being regressed. Associations of PCLs with hematologic measures are reported as standardized betas (to facilitate direct comparison of effect sizes across hematologic measures with different units of measure) and 95% CrIs. All analyses were conducted in R version 4.1.2 (https://cran.r-project.org/)
