# Supplementary material for: Childhood skeletal lesions common in prehistory are present in living forager-farmers and predict adult markers of immune function
Source: Sci Adv. 2025 Jul 16;11(29):eadw3697. doi: 10.1126/sciadv.adw3697 (PMC12266093; doi:10.1126/sciadv.adw3697)
Supplement: Supplementary file 1 — Supplementary Text Figs. S1 to S4 Tables S1 to S8 [file sciadv.adw3697_sm.pdf]

Science Advances
AAAS

Supplementary Materials for

Childhood skeletal lesions common in prehistory are present in living forager-farmers and predict adult markers of immune function

Amy S. Anderson *et al.*

Corresponding author: Amy S. Anderson, amy_anderson@eva.mpg.de;
Michael D. Gurven, gurven@anth.ucsb.edu

Sci. Adv. **11**, eadw3697 (2025)
DOI: 10.1126/sciadv.adw3697

This PDF file includes:

Supplementary Text
Figs. S1 to S4
Tables S1 to S8

Supplementary Text

Lesion morphology

Pitting/porosity was most common on the occipital bone (14.75%) and least common on the frontal bone (0.54%). When porous changes were present, the frontal and superior parietal bones invariably presented with fine, 'pinprick' porosity or pitting and were thus considered ambiguous cases, while larger more distinct foramina were seen on the posterior aspect of the parietal bones and on the squamous occipital bone. Cases of ambiguous fine porosity were noted almost exclusively among women, while larger more distinct foramina on the posterior parietal bones or occipital bones were more common among men, though this pattern was not statistically significant. While the limitations of CT visualization do lead to greater underreporting of lesions on the superior frontal and parietal bones (*1*), other studies of cranial cribra in South American populations have also reported the posterior parietal bones and central occipital squama in particular as the most common sites of vault lesions (*2–4*). Because radiological cross sections are more sensitive to expansive changes in the diploic space of the cranial vault than to surface porosity per se, clinical cases do not report much in the way of changes in the occipital squama, which does not have the same capacity as the frontal and parietal bones for expansion of the diploic space. In cases of acquired iron-deficiency anemia cranial changes are reported most often in the frontal squama, followed by the parietal bones (*5*).

Ectocranial pitting is widely interpreted by bioarchaeologists as an advanced stage of healing porosity. Mann and Hunt (*6*) note that it appears primarily in middle-aged adults. It can be difficult to distinguish pitting from porosity using traditional osteological methods of macroscopic observation, but CT provides a clear way to identify when apparent porosity on the cranial vault's outer table truly is the surface manifestation of perforations that extend into the diploe, and when porosity is more superficial. If ectocranial pitting is an advanced healing stage of cranial porosity acquired in early childhood, we should expect the proportion of porous cranial lesions that manifest as pitting rather than true porosity to grow increasing more common in older adults.

Ectocranial pitting is generally considered a more advanced stage of porous lesion healing than cranial vault porosity (*7*). Given this interpretation, we might expect that the proportion of lesions showing true porosity will decline with age and that ectocranial pitting will account for a correspondingly larger proportion of lesions among older individuals, but this was not the pattern observed in the current study. The proportion of pitting to porosity was roughly equivalent at all ages.

Assuming the lesions identified in Tsimane adults developed during childhood, several inferences can be drawn. First, ectocranial porosis is not associated with higher mortality risk for Tsimane adults older than 40 years. If it were, overall lesion frequency would decline with age. Second, the absence of an age-related shift from porosity to pitting suggests that individual variability may play a relatively large role in determining the retention of lesions over the lifespan and that complete remodeling (disappearance) of cranial vault lesions is unlikely at older ages.

Demographic Patterns

There are several possible explanations for cribra orbitalia's negative relationship with age. Lesions could be less observable at older ages because individuals with lesions face higher overall mortality risks than their non-lesioned peers and are thus less well represented in older cohorts. This is the interpretation typically made in archaeological contexts when cribra orbitalia is more common among younger individuals. Lesions in some individuals might also be obscured over a lifetime by skeletal remodeling. The rate at which lesions are obscured by remodeling has not yet been established, and skeletal remodeling likely renders lesions unobservable on CT scans faster than they become undetectable to direct observation of skeletal remains.

As of March 2024, 40 of the study participants (aged 51-88 years) had died. Of these, two are women with cribra orbitalia and four are men with CC. In light of this sample size, formal analysis of lesion-associated mortality risk is premature at this time but is planned for the future.

<u>Protocol for diagnosing respiratory infections, from the THLHP mobile medical team</u>

The diagnosis conducted by our medical brigade is primarily clinical, with support from a white blood cell differential count in cases of lower respiratory tract infections. Since our stay in each community ranges from 3 to 5 days, depending on the size of the population, we are unable to conduct long-term follow-up of cases.

When a participant arrives for a medical consultation, the diagnostic process begins with identifying the main symptom, typically fever or respiratory complaints. Fever is often a strong indicator of a potential infectious disease. The accompanying respiratory symptom helps localize the infection. However, not all patients present with fever, necessitating a differential diagnosis to rule out non-infectious conditions that may cause similar symptoms, such as rhinorrhea or cough.

For rhinorrhea (nasal discharge and sneezing), it is crucial to determine if it occurs in isolation or is accompanied by other symptoms. If the rhinorrhea is isolated and short-lived, it is likely due to allergic rhinitis, which is common in areas where trees are burned or cooking with wood occurs. However, if rhinorrhea is accompanied by fever, general malaise, or affects multiple family members, it is more likely to be a cold or possibly rhinopharyngitis if additional respiratory symptoms are present.

In adult patients with cough, we assess whether it is sporadic or follows a specific pattern. For instance, a nighttime cough when lying down may suggest a gastrointestinal issue, such as gastroesophageal reflux, which causes coughing due to aspiration of stomach contents during sleep. This assessment helps distinguish between respiratory and other causes.

Finally, during the physical examination, we follow a protocol to mobilize secretions and assess lung condition. If abnormal lung sounds, such as crackles or impaired ventilation, are detected, we perform a white blood cell differential count. Findings like neutrophilia and leukocytosis support a diagnosis of pneumonia, and these cases are referred for hospital treatment. Other respiratory infections are managed on an outpatient basis within the community.

Specific Symptoms and Signs for Diagnosis:

- Common Cold: Rhinorrhea, nasal congestion, sneezing.
- Sinusitis: Rhinorrhea, nasal voice, postnasal drip, pain upon pressing the affected sinuses. It is categorized as acute or chronic based on duration.

- Pharyngotonsillitis: Sore throat, fever, general malaise. Physical exam findings: congested oropharynx, enlarged tonsils, pus on tonsils, swollen neck lymph nodes, and normal lung sounds.
- Laryngitis: Hoarse voice, fever, general malaise. Normal lung sounds.
- Bronchiolitis: Cough and fever, with wheezing heard on auscultation.
- Bronchitis: Cough, low-grade fever, general malaise. Ronchi heard on auscultation.
- Pneumonia: Cough with purulent sputum (if expectoration is possible), high fever, chest pain, general malaise. Crackles heard on auscultation.

The main symptom of pulmonary tuberculosis is a persistent cough lasting more than two weeks. In many cases, we also consider the patient's history, especially if relatives have had tuberculosis or died from it. Our biotechnologist has been trained by specialists from the National Tuberculosis Control Program. Nevertheless, we refer suspected cases to San Borja Hospital for confirmation. If there is a strong suspicion of TB but the sputum test results are negative, the hospital sends the sample to a higher-level laboratory for biomolecular analysis (genexpert).

Tuberculosis, flow cytometry, and PCLs

Flow cytometry values were obtained in 2011-2013, whereas clinical diagnoses span 2002-2020. Roughly half of individuals who received a TB diagnosis during this time span were diagnosed in years following the blood draw from which flow cytometry values were obtained, while the other half were diagnosed and treated in years prior to flow cytometry measures. As a result, the data do not allow for a mechanistic unpacking of the relationship between CO, TB, and T cell population counts. No individuals were diagnosed with TB at the clinic visit from which their flow cytometry measures were obtained.

Diagnoses of the head and eyes

In addition to respiratory conditions, we examined associations with diagnoses of the eyes. Of course, lesions comprised of new bone formation in response to local inflammation, infection, or trauma need not be confined to childhood (*8*), though bone is more responsive to stimuli during growth and development. Because it is possible (though not probable) for PCLs to be caused at later ages by porous new bone, we tested associations between cribra orbitalia and medical conditions of the eyes and head. Localized responses should be considered particularly in the case of unilateral lesions. However, because orbital lesion presence on the scans can be determined with higher certainty than orbital lesion absence, a clear distinction between unilateral and bilateral cases could not be made using CT scans.

To assess whether localized eye conditions might be the primary cause of cribra orbitalia among the adults in this study, we tested whether orbital lesions were associated with eye infections or pterygium. Pterygium is a highly vascularized fleshy growth on the conjunctival surface of the eye. Risk factors for pterygium are high chronic levels of exposure to sun, wind, and dust (*9*). While it has not been clinically linked to orbital roof porosity, the increased ocular angiogenesis involved in its development and the high prevalence of pterygium in the study population make a possible connection worth investigating. Nevertheless, cribra orbitalia was not associated with any diagnosed eye conditions (Table S3).

Of the 372 individuals with observable status for both lesions, 39 were diagnosed with tuberculosis. Of these 39, CC was observed in 3 and CO was observed in 4. The relatively small number of lesion-positive, tuberculosis-positive cases is cause for caution in the conclusions that can be drawn about the association between these two conditions based on the present study.

Statistical methods: Additional details

Models for cribra orbitalia were run twice – first with ambiguous cases (n = 37) coded as lesion absence and then with ambiguous cases coded as lesion presence. Results were considered robust when both models returned consistent results. Only results of the first models (more conservative coding for lesion presence) are reported.

Raw values for all leukocyte population counts were logged prior to analysis. For models of cell counts from flow cytometry, for which fewer individuals had repeat measures, the group-level effect of individual identity was estimated only for the subset of individuals with flow cytometry data from multiple dates. The potential influence of current infection on hematological measures was tested by including a covariate for the residual white blood cell count, subtracting the leukocyte subtype being predicted by the model.

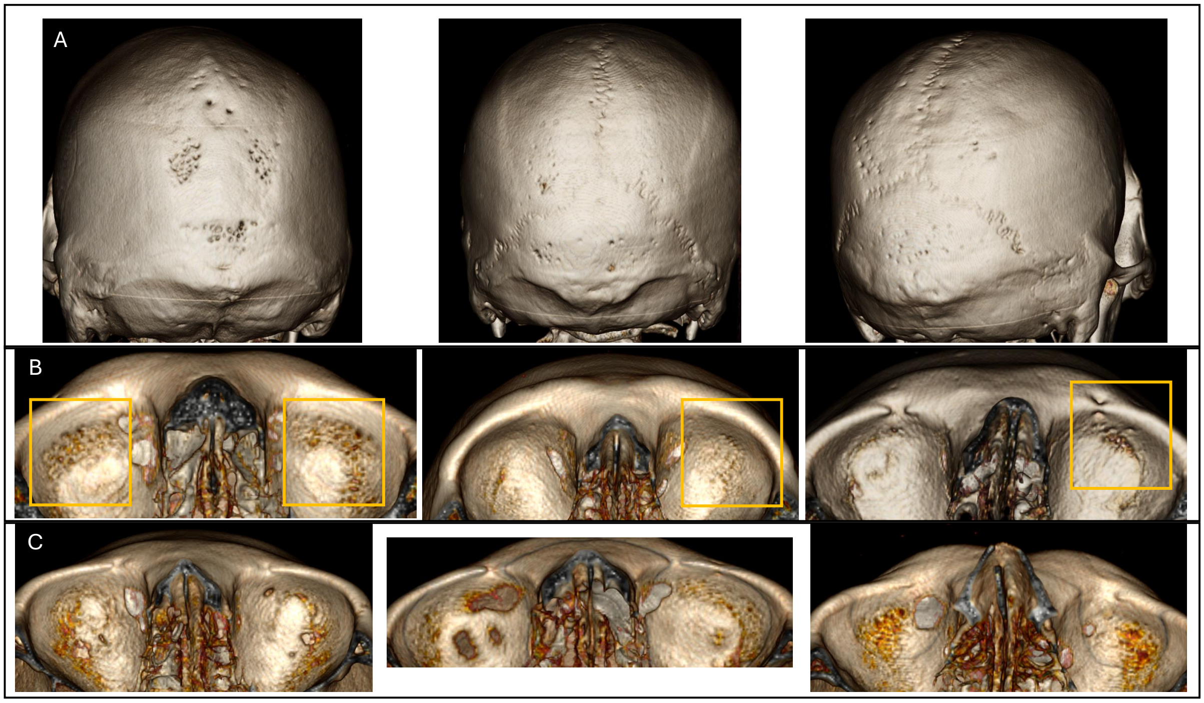

Fig. S1. 3D CT reconstructions from study participants. A) Additional examples of cribra cranii (CC). B) clearly visible CO (within yellow boxes). C) cases judged to be absent of CO. Absence of lesions can look like ambiguous cases when viewed as 3D reconstructions. In these cases, 3D porosity is not convincing, and 2D views do not show porosity.

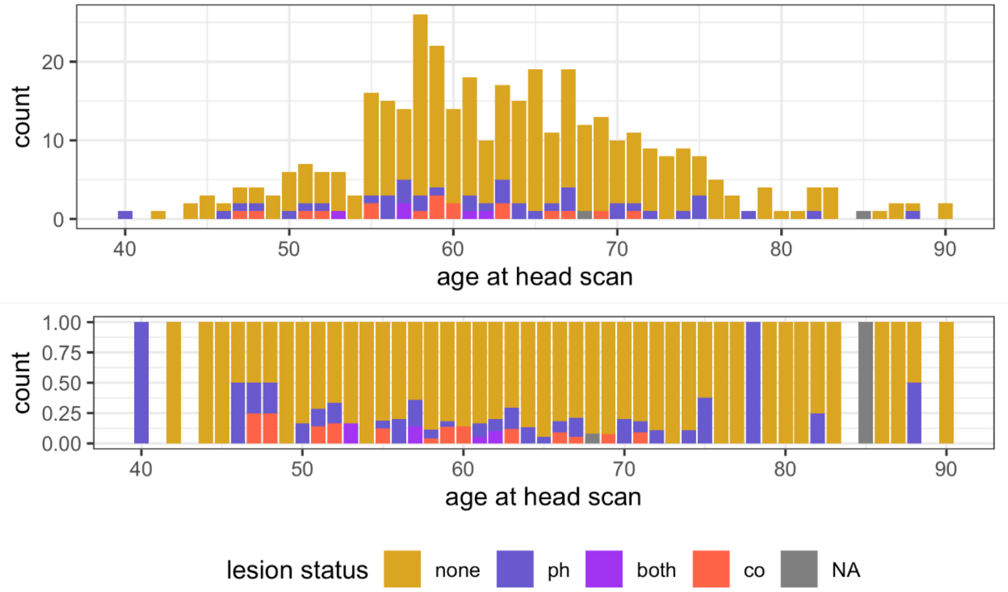

Fig. S2. Age distribution of porous cranial lesions in study sample. Top = raw counts, bottom = proportion of individuals at each age (single year intervals) with a given lesion status. NA = lesion status not observable from CT scan.

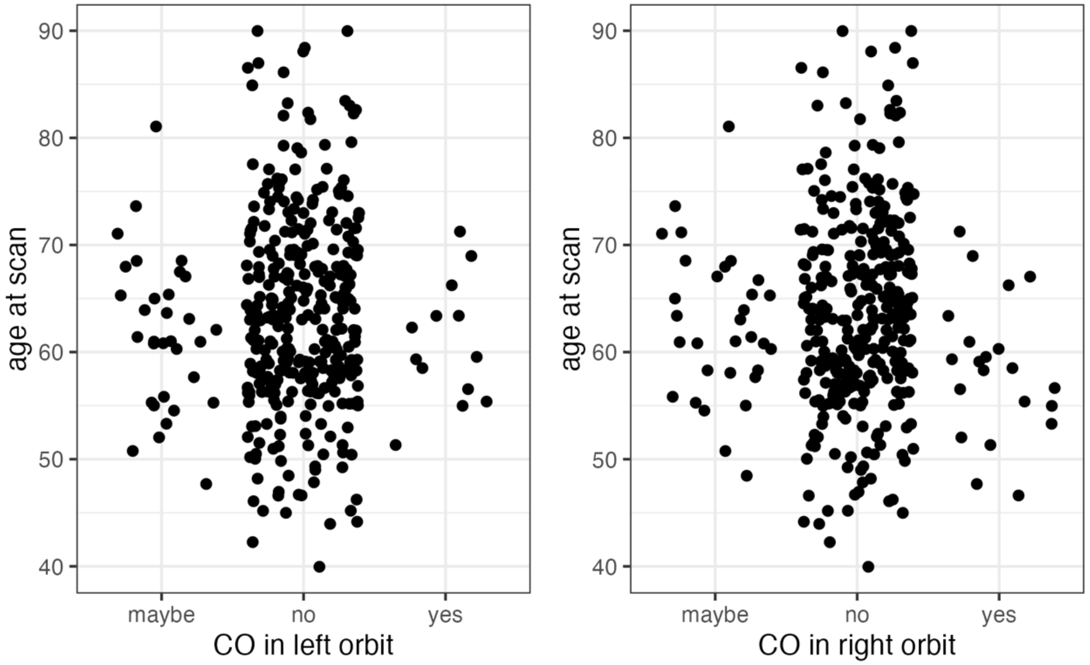

Fig. S3. Comparison of ages for each category of CO as evaluated from cranial CT scans. Ambiguous cases ("maybe") have similar ages to clearcut cases ("yes").

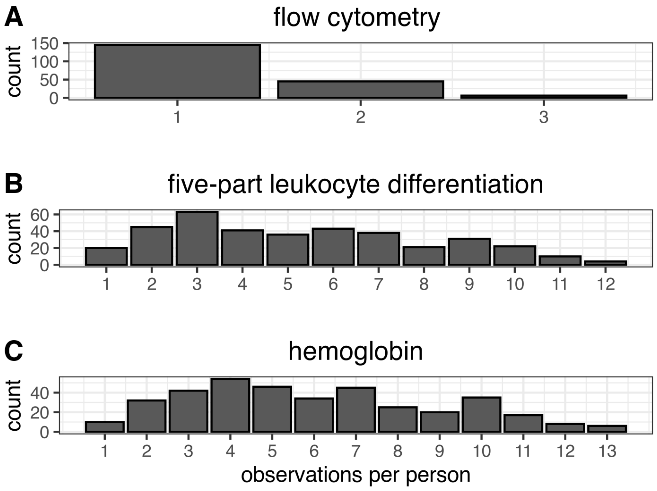

Fig. S4. Distribution of observations per person for blood biomarkers. A) Distribution of unique-date flow cytometry measures per person. B) Distribution of unique-date measures of five-part leukocyte differentiation per person. C) Distribution of hemoglobin observations available per person over the study period.

	Cribra orbitalia		Cribra cranii	
	Odds Ratio	95% CrI	Odds Ratio	95% CrI
(Intercept)	4.6	(0.18, 124.8)	0.18	(0.02, 1.9)
Age (years)	0.93	(0.88, 0.99)	0.99	(0.95, 1.02)
Male (vs. female)	0.72	(0.3, 1.7)	2.86	(1.45, 6.09)

Table S1. Presence of each lesion type as a function of age and sex. Odds ratios and 95% CrIs from logistic regressions; non-linear models of the same data are plotted in Figure 3 of the main text.

	OR	*lower 95% CrI*	*upper 95% CrI*
Upper Respiratory Infection			
intercept	0.397	0.191	0.817
age	0.96	0.947	0.972
CO	1.047	0.594	1.798
intercept	0.394	0.19	0.81
age	0.96	0.948	0.972
CC	0.953	0.645	1.38
Lower Respiratory Infection			
intercept	0.071	0.025	0.203
age	0.973	0.954	0.991
CO	0.794	0.296	2.007
intercept	0.094	0.028	0.315
age	0.96	0.938	0.981
CC	1.532	0.783	2.937
Tuberculosis			
intercept	0.001	0	0.007
age	1.091	1.052	1.133
CO	2.391	0.612	7.694
intercept	0.001	0	0.009
age	1.09	1.05	1.135
CC	0.458	0.11	1.538

Table S2. Effect of CO and CC on age-adjusted probability of being diagnosed with tuberculosis, upper respiratory infections, and lower respiratory infections. Multilevel logistic models for upper and lower respiratory infections include a random effect for individual participant.

		Cribra orbitalia (CO)		*Cribra cranii (CC)*	
Diagnosis	% of medical visits with diagnosis	OR	95% CrI	OR	95% CrI
pterygium	4.97	1.16	(0.47, 2.64)	0.78	(0.42, 1.4)
eye infection	2.06	1.33	(0.36, 4.22)	1.13	(0.48, 2.54)

Table S3. Association of orbital lesions with diagnosed conditions of the eyes. Odds ratios and 95% credible intervals, models adjusted for age and sex.

	No Tuberculosis diagnosis	*Symptomatic Tuberculosis*
CO^-	313	35
CO^+	19	4
CO^+ %	**6.1**	**11.4**
CC^-	287	37
CC^+	43	3
CC^+ %	**15**	**8.1**

Table S4. Correlation between PCLs and tuberculosis. Contingency table showing the number of people who did and who did not receive a tuberculosis diagnosis during the study period, and whether cribra orbitalia (CO) or cribra cranii (CC), despite the absence of visible hyperostotic changes) were visible on their cranial scan.

Outcome variable	**cribra orbitalia** *% difference in mean outcome with lesion*	*95% CrI*	**cribra cranii** *% difference in mean outcome with lesion*	*95% CrI*
hemoglobin (g/dL)	-0.86	(-4.21, 2.62)	-0.87	(-3.49, 1.72)
WBC	-6.02	(-13.45, 1.87)	-3.78	(-9.3, 2.49)
eosinophils	-11.06	(-29.5, 12.19)	-5.62	(-20.41, 11.62)
neutrophils	-9.07	(-18.53, 1.38)	-3.92	(-11.26, 4.14)
lymphocytes	1.57	(-9.01, 12.97)	0.68	(-6.92, 9.09)
natural killer cells	8.09	(-25.72, 57.11)	13.99	(-11.32, 47.32)
B cells	-32.05	(-52.42, -2.83)	-1.72	(-22.26, 24.21)
Total CD4 cells	-19.84	(-40.99, 7.32)	-10.97	(-26.98, 8.36)
naive CD4 cells	-51.72	(-74.48, -7.37)	1.34	(-34.47, 56.78)
nonnaive CD4 cells	-14.9	(-46.18, 35.05)	-7.46	(-31.39, 25.15)
Total CD8 cells	29.91	(-9.2, 86.86)	0.64	(-20.18, 26.68)
naive CD8 cells	18.18	(-30.04, 97.73)	7.32	(-24.61, 52.16)
nonnaive CD8 cells	43.98	(-19.93, 157.98)	6.38	(-27.16, 56.32)
CD4/CD8 ratio	-105.82	(-175.06, -37.69)	-25.35	(-69.92, 20.12)

Table S5. Values are plotted in the main text in Figure 5C. Percent difference in predicted values for a female at the sample median age in the presence of each cranial lesion, compared to her predicted values for each biomarker in the absence of cranial lesions. Bayesian multilevel linear regressions include fixed effects for sex and age at time of blood draw, and a random effect for individual participant.

	cribra orbitalia		cribra cranii	
outcome variable	*predicted % difference with lesion*	*95% CrI*	*predicted % difference with lesion*	*95% CrI*
hemoglobin	-1.03	(-4.46, 2.39)	-0.89	(-3.52, 1.67)
eosinophils	-8.72	(-27.68, 15.19)	-4.84	(-19.48, 12.05)
neutrophils	-8.44	(-17.43, 1.77)	-3.52	(-10.38, 3.59)
lymphocytes	4.67	(-5.61, 16.12)	2.2	(-5, 10.14)
natural killer cells	13.09	(-21.81, 63.63)	11.02	(-12.62, 41.88)
B cells	-30.69	(-51.55, -1.4)	-3.73	(-23.67, 21.86)
Total CD4 T cells	-17.88	(-39.04, 10.01)	-12.48	(-27.76, 6.57)
naive CD4 T cells	-48.99	(-73.81, -2.43)	-1.86	(-35.53, 51.27)
nonnaive CD4 T cells	-13.72	(-45.78, 34.94)	-8.37	(-32.11, 23.29)
Total CD8 T cells	31.59	(-7.97, 89.45)	-0.11	(-20.98, 26.08)
naive CD8 T cells	0.19	(-14.22, 17.61)	0.07	(-14.01, 17.11)
nonnaive CD8 T cells	47.6	(-19.41, 167.39)	5.33	(-28.46, 55.54)
CD4/CD8 T cell ratio	-100.73	(-170.76, -33.48)	-27.49	(-70.86, 16.5)

Table S6. Same models as previous two tables above, with an additional term to control for effects of current infection. This instead estimates the difference in baseline immune cell populations in the presence of cranial lesions.

	Estimate	*Lower 95% CrI*	*Upper 95% CrI*
Intercept	-0.3	-0.46	-0.15
age	0.01	0.01	0.01
CO	0	-0.12	0.13
CC	0.06	-0.02	0.14

Table S7. Results of a logistic mixed effects model predicting the probability of anemia (hemoglobin < 13 g/dL for males and < 12 g/dL for females, according to World Health Organization guidelines). The estimated effect of cribra orbitalia is centered on 0.

variable	*median*	*(5th, 95th) percentile*	*n (obs)*	*n (people)*	*median obs/person*	*SD obs/person*	*dates*
hemoglobin (g/dL)	13.2	(10.9, 15.4)	2248	374	6	2.97	2004 - 2020
total leukocytes (cells/uL)	9000	(5500, 14100)	2238	374	6	2.95	2004 - 2020
neutrophils (cells/uL)	4678	(2688.4, 8213.8)	1984	374	5	2.82	2004 - 2020
eosinophils (cells/uL)	1328	(318.3, 3552.7)	1984	374	5	2.82	2004 - 2020
lymphocytes (cells/uL)	2500	(1365.4, 4099.4)	1984	374	5	2.82	2004 - 2020
natural killer cells (cells/uL)	596.2	(230.7, 1313.2)	252	195	1	0.52	2011 - 2013
B cells (cells/uL)	320.5	(135.3, 752.8)	252	195	1	0.52	2011 - 2013
CD4 T cells (cells/uL)	552	(258.7, 1122.8)	253	196	1	0.52	2011 - 2013
naive CD4 cells (cells/uL)	66.8	(12, 277.7)	244	190	1	0.51	2011 - 2013
non-naive CD4 cells (cells/uL)	466.7	(226.4, 978.9)	244	190	1	0.51	2011 - 2013
CD8 T cells (cells/uL)	386.6	(152.9, 803.8)	253	196	1	0.52	2011 - 2013
naive CD8 cells (cells/uL)	186	(66.8, 504.7)	244	190	1	0.51	2011 - 2013
non-naive CD8 cells (cells/uL)	186.3	(65.7, 391.1)	244	190	1	0.51	2011 - 2013

Table S8. Descriptive statistics for continuous dependent variables.
